# Effects of bone morphogenetic protein 4 on TGF-*β*1-induced cell proliferation, apoptosis, activation and differentiation in mouse lung fibroblasts *via* ERK/p38 MAPK signaling pathway

**DOI:** 10.7717/peerj.13775

**Published:** 2022-07-27

**Authors:** Zhou Cai, Hua Guo, Jing Qian, Wei Liu, Yuanyuan Li, Liang Yuan, You Zhou, Ran Lin, Xiaohui Xie, Qiong Yang, Guoying Wu, Qiongqiong Li, Li Zhao, Fei Liu, Jian Wang, Wenju Lu

**Affiliations:** 1 State Key Laboratory of Respiratory Disease, National Clinical Research Center for Respiratory Disease, Guangzhou Institute of Respiratory Health, The First Affiliated Hospital of Guangzhou Medical University, Guangzhou, Guangdong, China; 2 Department of Pulmonary and Critical Care Medicine, Zhujiang Hospital, Southern Medical University, Guangzhou, Guangdong, China; 3 Key Laboratory of National Health Commission for the Diagnosis & Treatment of COPD, The People’s Hospital of Inner Mongolia Autonomous Region, Hohhot, Inner Mongolia, China

**Keywords:** Pulmonary fibrosis, BMP4, TGF-β1, Fibroblasts, Activation, Apoptosis

## Abstract

Fibroblasts, in particular myofibroblasts, are the critical effector cells in idiopathic pulmonary fibrosis (IPF), a deadly lung disease characterized by abnormal lung remodeling and the formation of “fibroblastic foci”. Aberrant activation of TGF-*β*1 is frequently encountered and promotes fibroblast proliferation, activation, and differentiation in pulmonary fibrosis. Hence, the inhibition of TGF-*β*1-induced lung fibroblast activation holds promise as a therapeutic strategy for IPF. The present study aimed to investigate the potential effect and underlying mechanisms of bone morphogenetic protein 4 (BMP4) on TGF-*β*1-induced proliferation, apoptosis, activation and myofibroblast differentiation of adult lung fibroblasts. Here, we demonstrated that BMP4 expression was significantly decreased in TGF-*β*1-stimulated mouse primary lung fibroblasts (PLFs). BMP4 inhibited proliferation and apoptosis resistance of TGF-*β*1-stimulated mouse PLFs. BMP4 suppressed TGF-*β*1-induced fibroblast activation and differentiation in mouse PLFs. We also found that BMP4 inhibited TGF-*β*1-induced ERK and p38 MAPK phosphorylation. Our findings indicate that BMP4 exerts its anti-fibrotic effects by regulating fibroblast proliferation, apoptosis, activation and differentiation *via* the inhibition of the ERK/p38 MAPK signaling pathway, and thus has a potential for the treatment of pulmonary fibrosis.

## Background

Idiopathic pulmonary fibrosis (IPF) is a chronic, devastating, and age-related interstitial lung disease that currently has no effective therapies (Raghu et al., 2011). Although the etiology and pathogenesis of IPF are not completely understood, fibroblasts that are involved in the repair and regenerative process, are perceived to play central roles (Sakai & Tager, 2013). In IPF lungs, fibroblasts migrate, proliferate, activate, and accumulate in “fibroblastic foci”, where is the prominent sites of superfluous extracellular matrix, and has been identified as the rising edge of active fibrosis (Gao et al., 2015; Scotton & Chambers, 2007). Therefore, inhibiting excessive fibroblast proliferation, activation and differentiation or promoting their apoptosis has considered as a promising method for therapy for IPF (Li et al., 2020).

Transforming growth factor (TGF)-*β*1 is a critical profibrotic cytokine answerable for the induction of fibroblasts proliferation, migration, activation and differentiation, and may play a crucial role in lung fibrosis (Guan et al., 2016a; Guan et al., 2016b). TGF-*β*1 level is increased in the lungs of animal models of lung fibrosis and humans with IPF (Guan et al., 2016a; Guan et al., 2016b; Kakugawa et al., 2004). Apart from facilitating proliferation, activation and apoptosis resistance, fibroblasts exposed to TGF-*β*1 can also differentiate to a profibrotic myofibroblast phenotype, which generates exaggerated amounts of ECM proteins and displays increased contractility (Zhao et al., 2016; Burgess et al., 2005). Hence, repressing TGF-*β*1 activation maybe a therapeutic target in IPF (Chen et al., 2013).

Bone morphogenetic proteins (BMPs) are multifunctional secreted growth factors that belong to the TGF-*β* superfamily (Zhang et al., 2014). They play key roles in regulating cell differentiation during embryonic lung development (Beyan et al., 2006; Bellusci et al., 1996). The balance between TGF-*β* and BMP signaling in the lung is of vital significance during developmental and regenerative processes (Costello et al., 2010; Dong et al., 2015). BMP4, a member of the BMPs family, has been reported to decrease proliferation but promote differentiation of fetal lung fibroblasts in the absence of any stimulation (Jeffery et al., 2005). BMP4 induces the proliferation of pulmonary epithelial cells (Shannon & Hyatt, 2004). BMP4 also induces growth arrest and apoptosis in multiple myeloma cell lines as well as in primary myeloma cells from patients (Westhrin et al., 2020). However, the direct effects of BMP4 on the TGF-*β*1-induced cell proliferation, apoptosis, activation and differentiation in adult lung fibroblasts remains unknown.

The purpose of this study was to investigate the effects of BMP4 on the TGF-*β*1-induced cell proliferation, apoptosis, activation and differentiation in adult mouse primary lung fibroblasts (PLFs) and to explore the underlying molecular mechanisms. We found that BMP4 is significantly down-regulated in TGF-*β*1-stimulated PLFs. BMP4 not only reduced TGF-*β*1-induced cell proliferation, activation and differentiation, but also decreased TGF-*β*1-induced apoptosis resistance in PLFs. We also demonstrated that BMP4 inhibited TGF-*β*1-induced adult lung fibroblast activation and differentiation *via* the ERK/p38 MAPK signaling pathway.

## Methods

### Chemicals and reagents

Murine recombinant BMP4 (Cat# 315-27) was purchased from PeproTech (Rocky Hill, NJ, USA), and mouse recombinant TGF-*β*1 (Cat# CK33) was purchased from novoprotein (SinoBio, Shanghai, China). Anti-proliferating cell nuclear antigen (PCNA, Cat# GB11010) and anti-Survivin (Cat# GB11177) antibodies were purchased from Servicebio (Wuhan, China). Anti-BMP4 antibody (Cat# YT7891) was purchased from Immunoway (TX, USA). Anti-fibroblast activation protein (FAP, Cat# A6349) and anti-Bcl-2 (Cat# A0208) antibodies were purchased from ABclonal Technology (Wuhan, China). Anti-p-ERK (Cat# 4370), Anti-ERK (Cat# 4695), anti-p-p38 MAPK (Cat# 4631), and anti-p38 MAPK (Cat# 8690) antibodies were purchased from Cell Signaling Technology (CA, USA). Anti-*α*-smooth muscle actin antibody (*α*-SMA, Cat# A2547) was purchased from Sigma-Aldrich (St. Louis, USA). Anti-*β*-actin antibody (Cat# ab2118), and the horseradish peroxidase-coupled secondary antibodies were purchased from Abcam Biotechnology (Cambridge, MA, USA). Other reagents were all purchased from GBCBIO Technologies Inc. (Guangzhou, China) unless otherwise indicated.

### Isolation of mouse primary lung fibroblasts

BMP4 heterozygous slie mutant mice (BMP4^+/ −^) with C57BL/6J background were provided by Jackson Laboratory (Bar Harbor, Maine, USA). The mice were housed in specific pathogen-free facilities with free access to laboratory chow and water, and the ambient temperature was maintained at 21–24 °C and humidity at 40%–60%. All experimental procedures were approved by the Animal Care and Use Committee of Affiliated First Hospital of Guangzhou Medical University (Acceptance number: 2021433).

PLFs were isolated from the mouse lungs (6∼8 weeks) by combining trypsin digestion and tissue adherent methods, according to previously described methods (Zhao et al., 2016). Briefly, mice were anesthetized by 1.2% Avertin solution. Then, the lung tissue of BMP4^+/ +^ (Wild-type, WT) or BMP4^+/ −^ mice at 6-8 weeks was cut into small pieces and washed with PBS until the liquid was clear. Trypsin (2.5 mg/ml) was added to digest the tissue for 15min. Lung tissue was moved into the culture flask, immersed in Dulbecco’s modified Eagle’s medium (DMEM; Gibco, Waltham, MA, USA) containing 10% FBS, and placed flasks overnight in a CO_2_ incubator at 37 °C. On the second day, the tissue blocks were washed with DMEM for 3 times, and the liquid was sucked away. The tissue block was placed evenly in the bottle. DMEM medium supplemented with 10% FBS was then added into the bottle, and the culture bottle was placed vertically in the cell incubator. After 3 h, gently put the bottle down, put the culture bottle in the incubator.

### Edu staining

For 5-ethynyl-2-deoxyuridine Edu assays, the proliferation ability of cells was examined with a BeyoClick™ EdU Cell Proliferation Kit with Alexa Fluor 555 (Cat#: C0075S; Beyotime, Haimen, China). The procedure was performed according to the manufacturer’s instruction. After Edu staining, cells were visualized using a fluorescence microscope (EVOS™ Auto 2, Invitrogen, WA, USA), and the percentage of Edu-positive cells was calculated as the number of Edu-positive cells out of the total number of cells (×100).

### Apoptotic cell measurement

WT or BMP4^+/ −^ PLFs were plated in a 6-well plate and treated with TGF-*β*1 and/or BMP4 for 48 h. Apoptotic PLFs were measured using Annexin V, fluorescein isothiocyanate (FITC) Apoptosis Detection Kit (R&D Systems, Minneapolis, MN, USA).

### Quantitative real-time PCR analysis (qRT-PCR)

Total RNA was extracted from PLFs using Trizol reagent (Invitrogen, Carlsbad, CA, USA) followed by reverse transcription a Color Reverse Transcription Kit (EZBioscience, USA). *qRT-PCR* was performed using with the above cDNA using qPCR SYBR Green Master Mix (Yeasen, Shanghai, China) according to the manufacturer’s protocol. Amplification was conducted using the following primers: 5′-TTGATACCTGAGACCGGGAAG- 3′ (forward) and 5′-ACATCTGTAGAAGTGTCGCCTC-3′ (reverse) for mouse BMP4, and 5′-GCAATTATTCCCCATGAACG-3′ (forward) and 5′-GGCCTCACTAAACCATCCAA-3′ (reverse) for 18s.

### Western blot

Western blot analysis was performed as described previously (Guan et al., 2019). Briefly, whole cell lysates of PLFs were extracted by radioimmune precipitation assay (RIPA) (Beyotime Biotechnology, Jiangsu, China) with protease and phosphatase inhibitors (Roche, USA). After determining the concentration of supernatant using a bicinchoninic acid (BCA) protein assay kit (Biocolor BioScience, Shanghai, China), protein samples of 50 µg per lane were loaded into 10% SDS-PAGE gels and transferred to PVDF membranes (Millipore, Billerica, MA, USA). All the blots were blocked with 5% non-fat milk in Tris-buffered saline containing 0.1% Tween-20 (TBST) for 1 h, and then incubated with corresponding primary antibodies at 4 °C overnight: anti-BMP4 (1:1,000), anti-*β*-actin (1:1,000), anti-PCNA (1:1,000), anti-Survivin (1:1,000), anti-Bcl-2 (1:1,000), anti-FAP (1:500), anti-*α*-SMA (1:1,000), anti-p-ERK (1:1,000), anti-ERK (1:1,000), anti-p-p38 (1:1,000), and anti-p38 (1:1,000). After incubation with corresponding secondary antibodies (1:5,000) with gentle shaking at room temperature for 1 h, the membranes were scanned by Tanon-5200 Imaging System (Tanon). Western blot bands were quantified using Image J by testing the band intensity for each group and normalizing it to *β*-actin as an internal control.

### Data and statistical analysis

Data analysis was performed using GraphPad Prism 6.0 (San Diego, CA, USA), and expressed as means  ± SEM from at least fourth independent experiments. Student’s *t*-test or one-way analysis of variance (One-way ANOVA) was performed to determine statistical significance. *p* < 0.05 indicated statistical significance.

## Results

### BMP4 is decreased in TGF-*β*1-stimulated mouse primary lung fibroblasts (PLFs)

**Figure 1:**
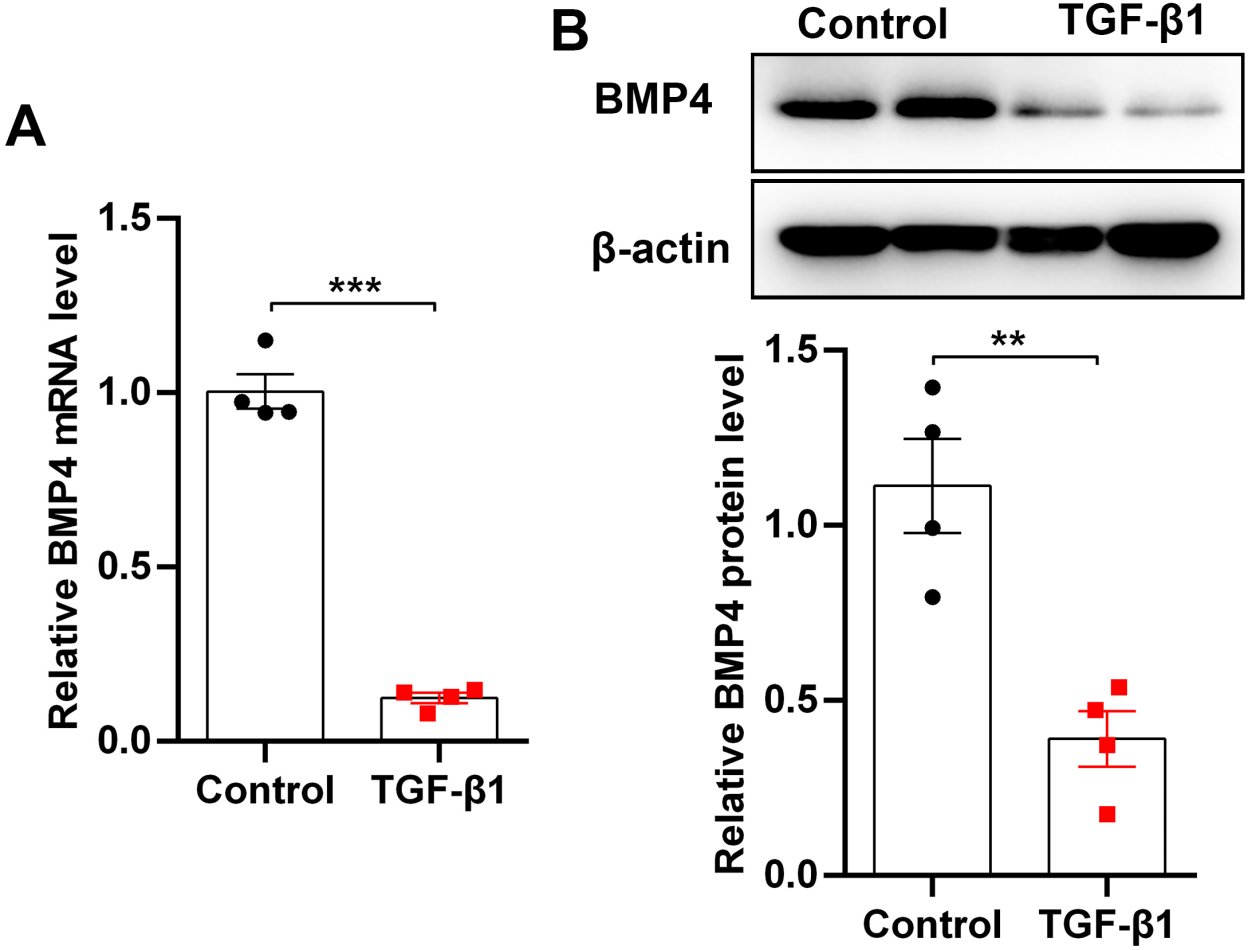
BMP4 was decreased in TGF- *β*1-stimulated mouse primary lung fibroblasts (PLFs). (A) qRT-PCR analysis of BMP4 mRNA levels in mouse PLFs stimulated with TGF- *β*1 (10 ng/ml) for 48 h. (B) Western blot analysis of BMP4 protein expression in mouse PLFs stimulated with TGF- *β*1 for 48 h. Data were expressed as mean ± SEM, ^**^p < 0.01; ^***^p < 0.001.

TGF-*β*1, an important fibrogenic factor, drives lung fibrosis by inducing fibroblast activation. By stimulation of mouse PLFs with TGF-*β*1, we found that BMP4 mRNA and protein levels were significantly decreased as demonstrated by qRT-PCR (Fig. 1A) and Western blot (Fig. 1B), indicating that BMP4 deficiency is positively associated with fibroblast activation and lung fibrosis.

### BMP4 inhibits TGF-*β*1-induced cell proliferation in mouse primary lung fibroblasts (PLFs)

**Figure 2:**
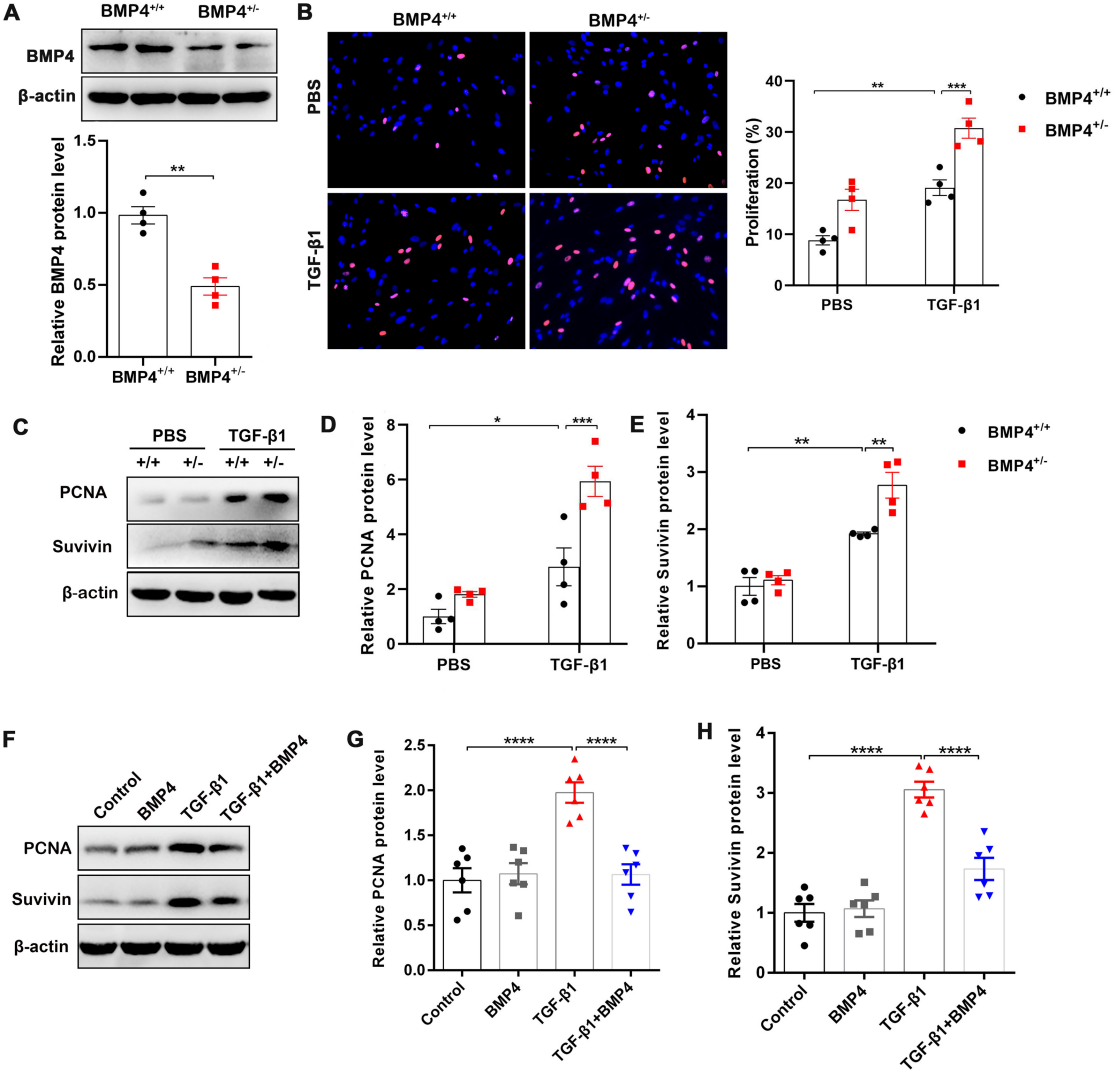
BMP4 inhibited TGF- *β*1-induced proliferation of mouse PLFs. (A–C) Western blot analysis of PCNA and Survivin in total cell lysates of PLFs from BMP4^+/ +^ and BMP4^+/−^ mice treated with TGF- *β*1 (10 ng/ml, 48 h). *β*-actin was used as a loading control. (D–E) Western blot analysis of PCNA and Survivin protein expressions in total cell lysates of WT PLFs treated with TGF- *β*1 (10 ng/ml, 48 h) and/or BMP4 (20 µM). *β*-actin was used as a loading control. Data were expressed as mean ± SEM, ^**^p < 0.01; ^****^p < 0.0001.

Fibroblast/activated fibroblast proliferation occurs at the initial stage of tissue repair in response to injury, and the apoptosis of fibroblasts is critical to restore normal tissue architecture (Lorena et al., 2002). We isolated mouse PLFs from WT or BMP4^+/ −^ mice, and found BMP4 level was reduced approximately 50.2% in BMP4^+/ −^ PLFs (Fig. 2A). To investigate whether BMP4 affects the proliferation of fibroblasts/activated fibroblasts, mouse PLFs from WT or BMP4^+/ −^ were treated with TGF-*β*1 for 48 h and Edu staining was performed. The results showed that a significant enhancement of fibroblast proliferation was observed in TGF-*β*1-treated cells, which was then further increased by BMP4 haploinsufficiency (Fig. 2B). In addition, the expression levels of PCNA and Survivin, two critical cell proliferation proteins, were measured by Western blot. As shown in Figs. 2C–2E, treatment with TGF-*β*1 caused an increased PCNA and Survivin expressions, whereas the TGF-*β*1-induced upregulation of PCNA and Survivin were also further increased by BMP4 haploinsufficiency. In line with this finding, the TGF-*β*1-induced upregulation of PCNA and Survivin was significantly hampered by addition of exogenous BMP4 (Figs. 2F–2H). These data suggest that BMP4 can inhibit TGF-*β*1-induced fibroblast/myofibroblast proliferation.

### BMP4 promotes cell apoptosis in TGF-*β*1-stimulated mouse primary lung fibroblasts (PLFs)

**Figure 3:**
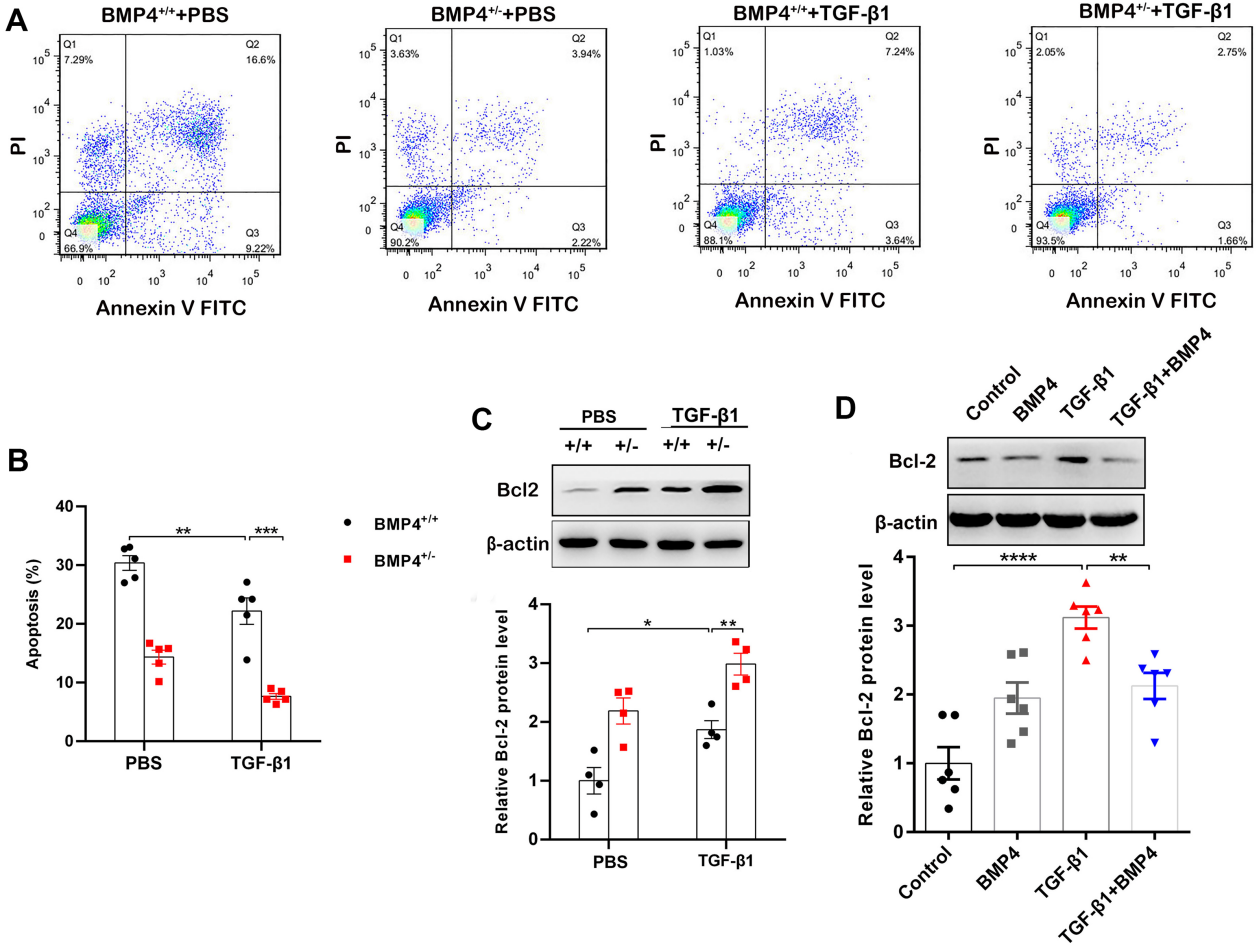
BMP4 promoted cell apoptosis in TGF- *β*1-stimulated mouse PLFs. (A) PLFs from BMP4^+/ +^ and BMP4^+/−^ mice were treated with TGF- *β*1 (10 ng/mL) for 48 h. The cells were double-stained with Annexin V-FITC and PI, and then the cellular apoptosis was determined by flow cytometry. (B) The ratio of apoptotic cells was statistically analyzed. (C) Western blot analysis of Bcl-2 in total cell lysates of PLFs from BMP4^+/ +^ and BMP4^+/−^ mice treated with TGF- *β*1 (10 ng/ml, 48 h). *β*-actin was used as a loading control. (D) Western blot analysis of Bcl-2 protein expression in total cell lysates of WT PLFs treated with TGF- *β*1 (10 ng/ml, 48 h) and/or BMP4 (20 µM). *β*-actin was used as a loading control. Data were expressed as mean ± SEM, ^*^p < 0.05; ^**^p < 0.01; ^***^p < 0.001.

Decreased apoptosis in fibroblasts/myofibroblasts promote the formation of fibrotic lesions in IPF (Fattman, 2008). Flow cytometry was therefore used to investigate whether BMP4 affects apoptosis in PLFs. The results showed that BMP4 haploinsufficiency decreased cellular apoptosis in both unstimulated and TGF-*β*1-stimulated fibroblasts (Figs. 3A, 3B). Western blot showed that Bcl-2, an anti-apoptotic protein, was also significantly increased by TGF-*β*1, which was further enhanced by BMP4 haploinsufficiency (Fig. 3C). Likewise, the TGF-*β*1-induced upregulation of Bcl-2 was also reduced by the introduction of exogenous BMP4 (Fig. 3D). Taken together, these results suggest that BMP4 promotes apoptosis in TGF-*β*1-stimulated lung fibroblasts.

### BMP4 suppresses TGF-*β*1-induced fibroblast activation and myofibroblast differentiation in mouse primary lung fibroblasts (PLFs)

**Figure 4:**
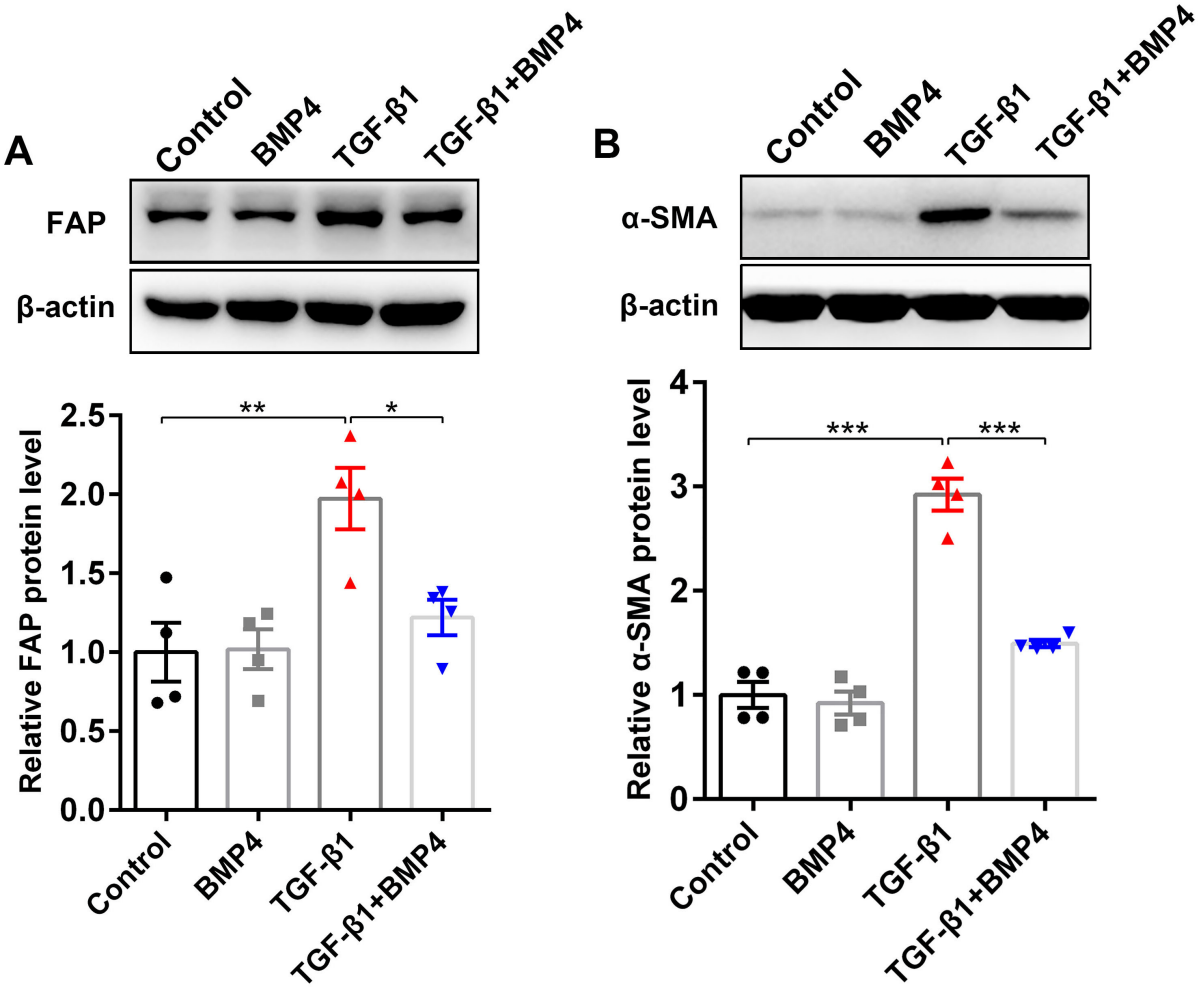
BMP4 suppressed TGF- *β*1-induced fibroblast activation and differentiation in mouse PLFs. PLFs were incubated with TGF- *β*1 (10 ng/ml) in the absence or presence of BMP4 (20 µM) for 48 h. Western blot assay was used to detect FAP (A) and *α*-SMA (B) protein expression. *β*-actin was used as a loading control. Data were expressed as mean ± SEM, ^*^p < 0.05; ^**^p < 0.01; ^***^p < 0.001.

FAP, a cell surface serine protease, is upregulated on a subset of activated fibroblasts, including fibroblasts in IPF (Kimura et al., 2019). Here, we demonstrated that TGF-*β*1 induced an upregulation of FAP in PLFs, consistent with fibroblast activation (Fig. 4A). The effect on TGF-*β*1-induced FAP enhancement was blocked by BMP4 supplement (Fig. 4A), suggesting the inhibitory effects of BMP4 on fibroblast activation. Fibroblasts exposed to TGF-*β*1 can differentiate into myofibroblasts that oversynthesize collagen and other ECM proteins (Hettiarachchi et al., 2020; Guan et al., 2016a; Guan et al., 2016b). The results showed that BMP4 rescued the TGF-*β*1-induced upregulation of *α*-SMA levels (Fig. 4B), indicating the inhibitory effects of BMP4 on myofibroblast differentiation.

### BMP4 downregulates TGF-*β*1-activated ERK1/2/p38 MAPK signaling pathway in primary lung fibroblasts (PLFs)

**Figure 5:**
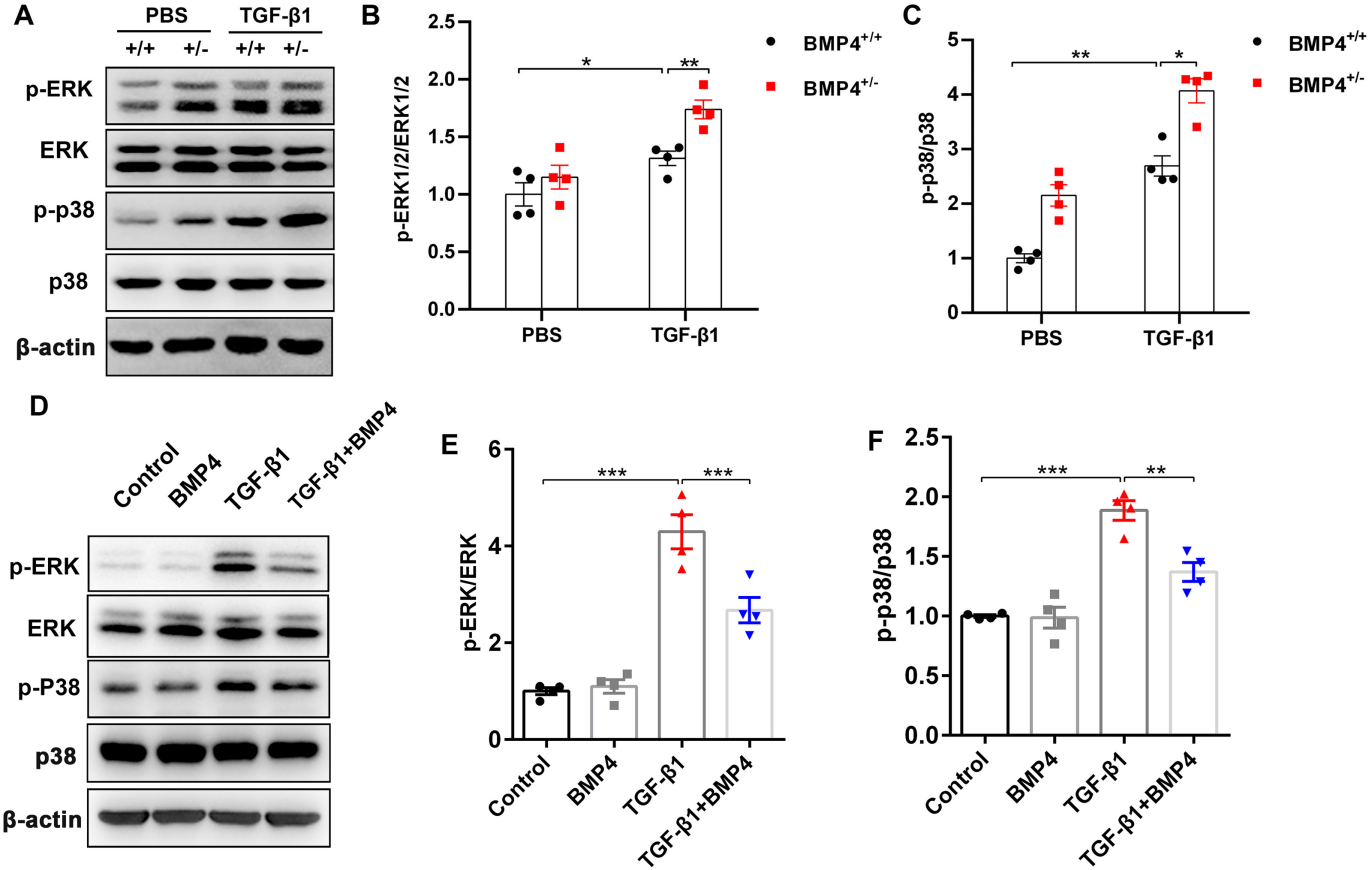
BMP4 downregulated TGF- *β*1-activated ERK/p38 MAPK signaling in PLFs. (A) Western blot analysis of phosphorylated and total ERK or p38 in total cell lysates of PLFs from BMP4^+/ +^ and BMP4^+/−^ mice treated with TGF- *β*1 (10 ng/ml, 48 h). *β*-actin was used as a loading control. (B) Western blot analysis of phosphorylated and total expression of ERK or p38 in total cell lysates ofPLFs treated with TGF- *β*1 (10 ng/ml) and/or BMP4 (20 µM). *β*-actin was used as a loading control. Data were expressed as mean ± SEM, ^*^p < 0.05; ^**^p < 0.01; ^***^p < 0.001.

TGF-*β*1 can induce fibroblast activation and differentiation *via* the ERK/p38 MAPK signaling pathway (Nie et al., 2017; Ng et al., 2019). Thus, we examined whether BMP4 mediated TGF-*β*1-induced fibroblast activation and differentiation *via* the ERK/p38 MAPK signaling pathway. Western blot assay demonstrated that the p-ERK and p-p38 MAPK were markedly increased in TGF-*β*1-stimulated mouse PLFs, when compared to control cells. Nevertheless, BMP4 haploinsufficiency significantly enhanced the TGF-*β*1-induced the phosphorylation of ERK and p38 MAPK (Figs. 5A–5C). Correspondingly, the TGF-*β*1-induced upregulation of phosphorylated ERK and p38 MAPK were significantly repressed by BMP4 administration (Figs. 5D–5F). These findings indicate that BMP4 effectively blocked TGF-*β*1-activated ERK/p38 MAPK signaling, which are involved in the attenuation of fibroblast activation and differentiation in lung fibroblasts.

## Discussion

The purpose of this study was to explore whether BMP4 can regulate TGF-*β*1-induced cell proliferation, apoptosis, activation and differentiation of mouse lung fibroblasts. Our result showed that BMP4, which is decreased by TGF-*β*1 stimulation, apparently attenuated TGF-*β*1-induced cell proliferation, apoptosis resistance, activation and differentiation in PLFs. Additionally, we established that BMP4 exerts its effect on PLFs by inhibiting the ERK/p38 MAPK signaling pathway. This is the first study to demonstrate the effects of BMP4 on TGF-*β*1-induced cell proliferation, apoptosis, activation and differentiation within adult mouse PLFs, which may provide a novel therapy for IPF.

IPF is characterized by excessive accumulation of active fibroblasts/myofibroblasts producing a mass of collagens in the lung that destroy its architecture and function (Sakai & Tager, 2013). These active fibroblasts/myofibroblasts cluster in “fibroblastic foci” of IPF lungs that has identified to be the primary edge of active fibrosis. Although the etiology and pathogenesis of IPF remain largely unknown, active fibroblasts/myofibroblasts, have been considered to be the most important players in this chronic disease (Sakai & Tager, 2013). Lung fibroblasts undergo a TGF-*β*1-dependent cell proliferation, apoptosis resistance and differentiation and thus acquire the ability to produce collagens, promoting IPF (Gao et al., 2015). Hence, TGF-*β*1 was widely used as a stimulator to trigger cell proliferation, apoptosis resistance, activation and differentiation in fibroblasts (Guan et al., 2016a; Guan et al., 2016b). Dysregulated fibroblast proliferation in IPF lungs has received considerable attention (Ryu et al., 2014). Previous study demonstrated that BMP4 inhibits proliferation of human fetal lung fibroblasts (Jeffery et al., 2005). However, the direct effects of BMP4 on TGF-*β*1-induced cell proliferation in adult lung fibroblasts remain uncertain. In this study, we demonstrated that treatment with BMP4 attenuated, whereas BMP4 haploinsufficiency aggravated, TGF-*β*1-induced cell proliferation in mouse PLFs. In conformity to our study, similar results were observed in aortic and pulmonary vascular smooth muscle cells (Morrell et al., 2001). Myofibroblasts, the critical effector cells in various fibrotic diseases including IPF where they have increased contractility and cause turgescent collagen deposition, mostly originate from the differentiation of resident mesenchymal cells (Hinz et al., 2012). Around 50% of myofibroblasts are derived from resident interstitial lung fibroblasts (King, Pardo & Selman, 2011). Thus, we investigated whether BMP4 affects TGF-*β*1-induced myofibroblast differentiation. In this study, we demonstrated that treatment with BMP4 also attenuated TGF-*β*1-induced myofibroblast differentiation in mouse PLFs. Conversely, BMP4 was demonstrated to upregulate *α*-SMA expression in human fetal lung fibroblasts, indicating that BMP4 can induce pulmonary myofibroblast differentiation of fetal lung fibroblasts (Jeffery et al., 2005). These contradictory results may be explained by the facts that BMP4 can affect myofibroblast differentiation differently according to cell type, age, external stimulus and stress levels, which indicates that metabolic capacity determines the cellular vulnerability to BMP4 deficiency in cells. Moreover, these activated fibroblasts can be distinguished from nonpathogenic fibroblasts by their expression of FAP, a membrane-spanning protein that is critical for collagen remodeling (Acharya et al., 2006). The TGF-*β*1-induced fibroblast activation was also inhibited by BMP4. Taken together, this result suggests that BMP4 inhibits TGF-*β*1-induced fibroblast activation and differentiation in mouse PLFs.

In addition to promoting fibroblast proliferation, activation and differentiation, TGF-*β*1 provides protection against apoptosis (Phan, 2002). Hence, TGF-*β*1 is important for the emergence of the myofibroblast and its survival against apoptotic stimuli, which suggests TGF-*β*1 may be critical in lung fibrosis by right of this new ability to promote myofibroblast survival by preventing the myofibroblast from undergoing apoptosis. In this study, we also assessed the role of BMP4 in apoptosis of fibroblasts and myofibroblasts/activated fibroblasts. We found that BMP4 also induced their apoptosis by modulating the levels of apoptosis regulatory proteins, which is in agreement with a previous study that BMP4 mediates cardiac hypertrophy, and apoptosis in experimentally pathological cardiac hypertrophy (Sun et al., 2013). This is very important because reduced apoptosis of fibroblasts and myofibroblasts contributes to the formation of fibrotic lesions and apoptosis induction in these cells can attenuate the severity of IPF (Fattman, 2008). These results imply that BMP4 can suppress proliferation of cultured PLFs by inducing apoptosis. Caspases are key molecules for the transduction and ejection of the apoptotic signals; further research is required to investigate whether BMP4 induces caspase-dependent apoptosis in PLFs. These findings indicate that BMP4 may attenuate BLM-induced lung fibrosis by modulating fibroblast and myofibroblast activities, which deserves to be studied in the future.

The mechanism by which BMP4 attenuated TGF-*β*1-induced fibroblast activation and differentiation remains obscure. Increasing evidence shows a critical role for TGF-*β*1 in fibroblasts. As members of TGF-*β* superfamily, both TGF-*β* and BMP4 bind to their receptors to activate downstream pathways, including the ERK and p38 MAPK signaling pathways, which were supposed to contribute to the induction of fibroblast proliferation, activation and differentiation after stress stimuli (Sun et al., 2017; Nie et al., 2017; Ng et al., 2019). The protective effect of BMP4 in lung fibroblast was also associated with the inactivation of the ERK and p38 MAPK pathways, which was in line with a previous finding (Ma et al., 2019). These observations support the notion that BMP4 represents a fatal factor that suppresses the development of lung fibrosis. However, Jeffery et al. (2005) reported that the activation of ERK appeared to occur in a biphasic manner, with an initial decrease in protein expression from 15 to 90 min and an activation after 120 min in human fetal lung fibroblasts. While the activation of p38 MAPK appeared to occur in a biphasic manner, with an initial elevation in protein level at 5 min and decreasing after 30 min and a second activation after 90 min in these fetal lung fibroblasts. These inconsistent studies also demonstrated that BMP4 may activate and/or inhibit different signaling pathways in various cells, according to cell type, age, external stimulus, and stress levels.

## Conclusions

BMP4 attenuated TGF-*β*1-induced proliferation, apoptosis resistance, and myofibroblast differentiation within adult lung fibroblast by inhibiting TGF-*β*1-activated ERK/p38 MAPK signaling. We propose that BMP4 is a promising pharmacological tool for the therapy of IPF.

## Statements

A protocol including the research question, key design features, and analysis plan was prepared before the study; however, it was not registered.

##  Supplemental Information

10.7717/peerj.13775/supp-1 Supplemental Information 1 The ARRIVE guidelines 2.0: author checklist

10.7717/peerj.13775/supp-2 Supplemental Information 2 All the uncropped original western blots

10.7717/peerj.13775/supp-3 Supplemental Information 3 Original data

## Additional Information and Declarations

### Data Availability

The following information was supplied regarding data availability: All the uncropped original western blots and raw data are available in the Supplemental Files.
