# Supplementary material for: Effects of bone morphogenetic protein 4 on TGF-β1-induced cell proliferation, apoptosis, activation and differentiation in mouse lung fibroblasts via ERK/p38 MAPK signaling pathway
Source: PeerJ. 2022 Jul 27;10:e13775. doi: 10.7717/peerj.13775 (PMC9338752; doi:10.7717/peerj.13775)

Western blot presented in this manuscript are listed as follows:

Figure 1B:

BMP4:

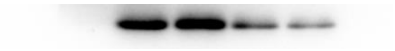

β-actin:

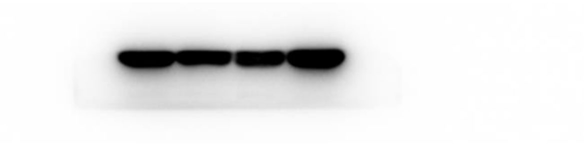

Figure 2A:

BMP4:

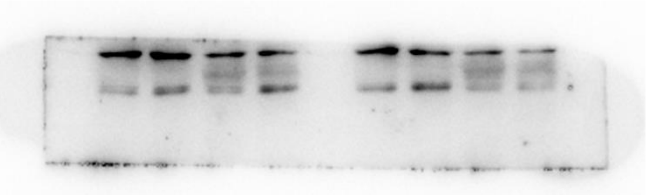

β-actin:

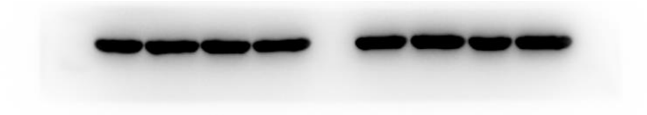

Figure 2C:

Suvivin:

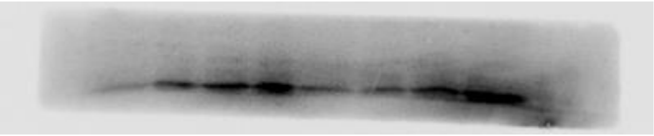

PCNA:

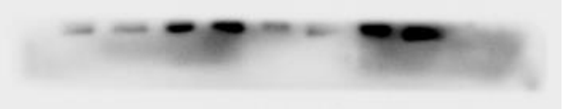

β-actin:

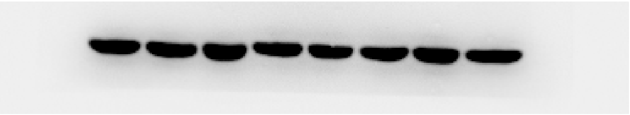

Figure 2F:

PCNA:

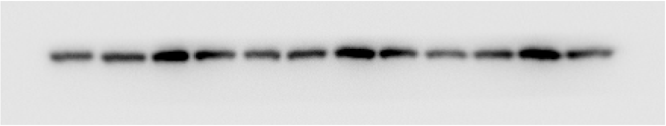

Suvivin:

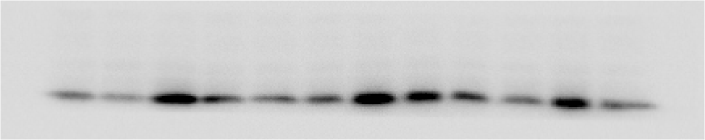

β-actin:

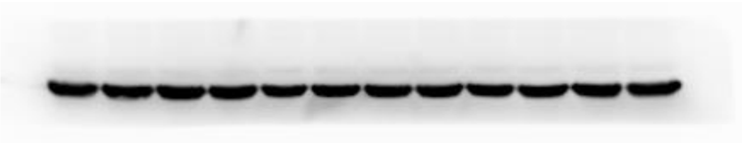

Figure 3C:

Bcl-2:

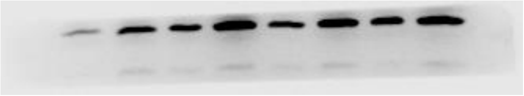

β-actin:

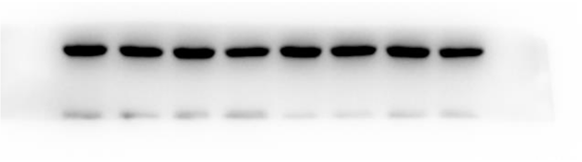

Figure 3D:

Bcl-2:

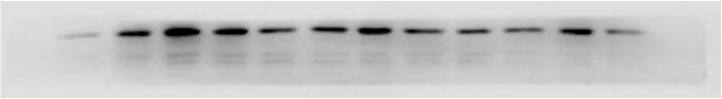

β-actin:

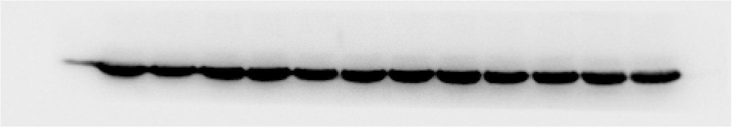

Figure 4A:

FAP:

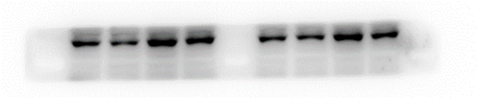

β-actin:

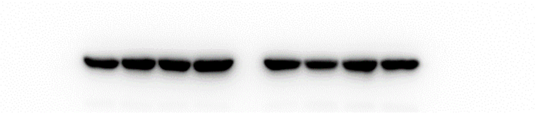

Figure 4B:

α-SMA:

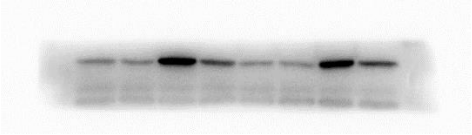

β-actin:

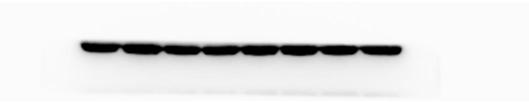

Figure 5A:

p-ERK:

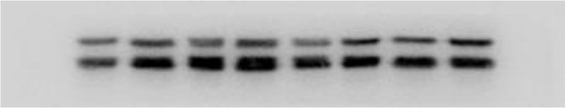

ERK:

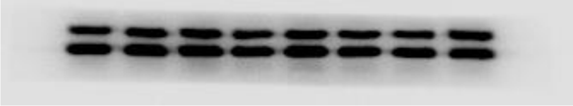

p-p38:

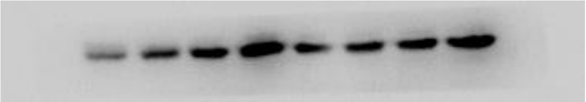

P38:

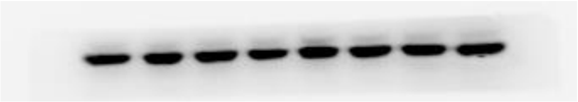

β-actin:

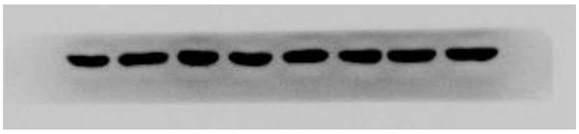

Figure 5D:

p-ERK:

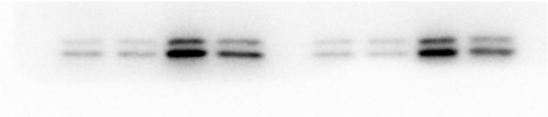

ERK:

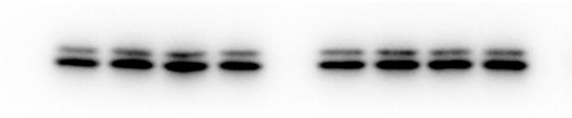

p-p38:

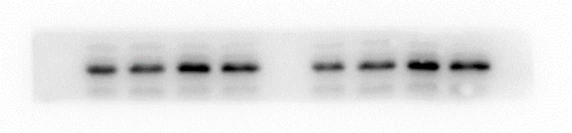

P38:

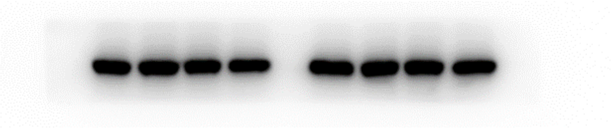

β-actin:

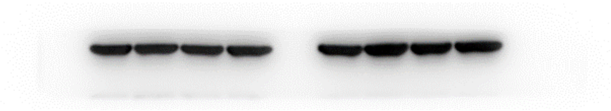

Supplement: Supplemental Information 2 [file peerj-10-13775-s002.pdf]
